# 2258. "Identification of Respiratory Viral Pathogens Using Real-Time PCR Was Not Associated with Reduced Antibiotic Use During the COVID-19 Pandemic, but Displayed Viral-Viral Interactions"

**DOI:** 10.1093/ofid/ofad500.1880

**Published:** 2023-11-27

**Authors:** Asif Khan, Daynha P Marti Ojeda, Richard A Murphy, Joel Elzweig, Victoria W Lacasse, Suzette Rovelsky

**Affiliations:** Dartmouth Hitchcock Medical Center, Lebanon, New Hampshire; Veterans Affairs, White River Junction, Vermont; White River Junction VA Medical Center, White River Junction, Vermont; White River Junction VA Medical Center, White River Junction, Vermont; White River Junction VA Healthcare System, White River Junction, Vermont; White River Junction VA Medical Center, White River Junction, Vermont

## Abstract

**Background:**

We aimed to investigate the relationship between SARS-CoV-2 and other circulating respiratory pathogens. A secondary objective was to assess the correlation between respiratory pathogen detection and antibiotic use at a single medical center during the COVID-19 pandemic.

**Figure:**
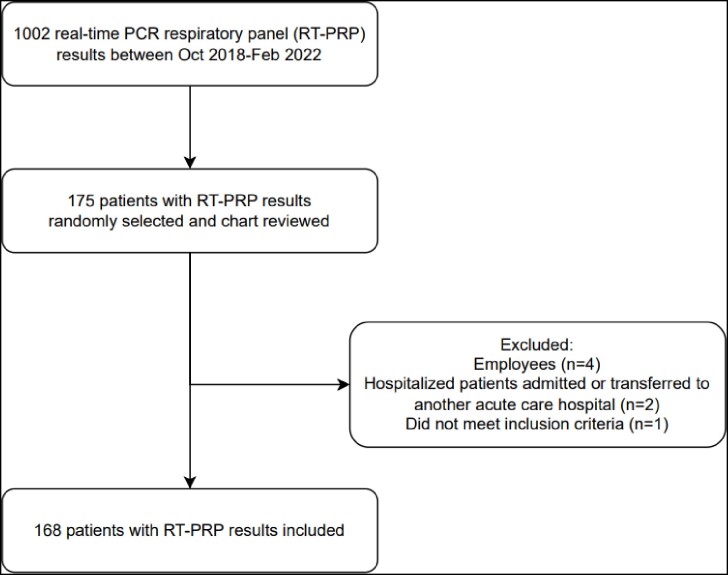

**Table 1.**
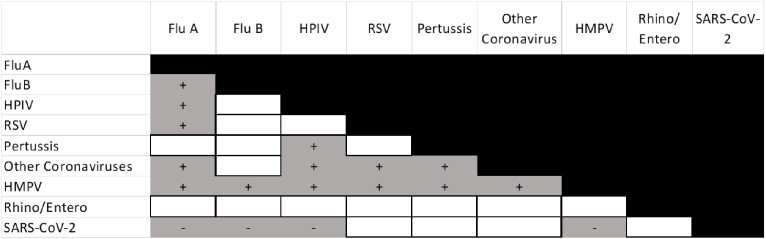
Negative and positive interactions among viruses at population scale (n=883)

Significant unadjusted correlations from bivariate cross-correlation analysis applying Spearman’s rank method to monthly viral infection prevalence are shown in gray, with negative and positive correlations indicated by − and +, respectively, and noncorrelated virus pairs in white.

**Methods:**

For viral-viral interaction analysis, data were collected from infection control surveillance reports on all positive respiratory viral pathogen tests (e.g., antigen, PCR) conducted between October 2018 and February 2022. Real-time PCR respiratory panel (RT-PRP) results (negative or positive) were extracted from the VA Corporate Data Warehouse for inpatients and outpatients. A random subset of patients (15-20%) was selected for chart review. Baseline characteristics, signs and symptoms, laboratory results, imaging interpretation, and admission and discharge diagnoses were collected. Spearman's rank correlation was employed to identify viral-viral interactions. Chart review data were analyzed using Wilcoxon-rank sum tests and Fisher's exact test.

**Table 2.**
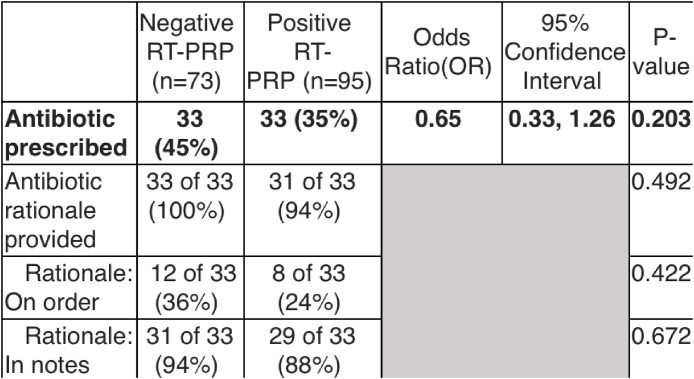
Correlations between results of RT-PRP result and antibiotics prescribed.

**Results:**

A total of 883 positive surveillance tests were analyzed for viral-viral interactions. A significant negative relationship was identified between SARS-CoV-2 and the prevalence of 43% of other respiratory pathogen types. Among 1,002 RT-PRP results, 168 patients with an RT-PRP result were chart reviewed. Although numerically a smaller proportion of patients with positive RT-PRP – compared to negative RT-PRP – received antibiotics this difference did not reach statistical significance.

**Conclusion:**

This project highlights the diverse influence of SARS-CoV-2 on the prevalence of other respiratory pathogens. No significant impact was detected on antibiotic use of a positive RT-PRP result. A positive RT-PRP result represents only one factor leading to the utilization of antibiotics, with other factors including clinician experience, symptom duration and severity, immunocompetency, and imaging. Based on these findings, we aim to enhance the integration of viral pathogen identification into antibiotic stewardship efforts.

**Disclosures:**

**All Authors**: No reported disclosures

